# Synthesis, crystal structure and thermal properties of di­bromido­bis­(2-methyl­pyridine *N*-oxide-κ*O*)cobalt(II)

**DOI:** 10.1107/S2056989024000252

**Published:** 2024-01-12

**Authors:** Christian Näther, Inke Jess

**Affiliations:** a Institut für Anorganische Chemie, Universität Kiel, Germany; Universität Greifswald, Germany

**Keywords:** crystal structure, powder X-ray diffraction, synthesis, cobalt bromide, 2-methyl­pyridine *N*-oxide, differential thermoanalysis, differential scanning calorimetry

## Abstract

Reaction of CoBr_2_ with 2-methyl­pyridine *N*-oxide in *n*-butanol leads to the formation of the title compound, [CoBr_2_(C_6_H_7_NO)_2_] or [CoBr_2_(2-methyl­pyridine *N*-oxide)_2_]. Its asymmetric unit consists of one Co^II^ cation as well as two bromide anions and two 2-methyl­pyridine *N*-oxide coligands in general positions. The Co^II^ cations are tetra­hedrally coordinated by two bromide anions and two 2-methyl­pyridine *N*-oxides, forming discrete complexes. In the crystal structure, these complexes are linked predominantly by weak C–H⋯Br hydrogen bonding into chains that propagate along the crystallographic *a*-axis. Powder X-ray diffraction (PXRD) measurements indicate that a pure phase was obtained. Thermoanalytical investigations prove that the title compound melts before decomposition; before melting, a further endothermic signal of unknown origin was observed that does not correspond to a phase transition.

## Abstract 2

The crystal structure of the title compound consists of discrete tetra­hedral complexes that are linked by weak inter­molecular hydrogen bonding. Upon heating, melting before decomposition is observed.

## 1. Chemical context

Numerous transition-metal halide coordination compounds have been reported in the literature (Peng *et al.*, 2010 ▸). Most of these compounds are characterized by metal halide substructures such as, for example, mono- and dinuclear units, chains or layers, that can be further linked by bridging ligands into 1-, 2- and 3-D networks (Peng *et al.*, 2010 ▸; Näther *et al.*, 2007 ▸). We are especially inter­ested in the thermal properties of such compounds because we have found that compounds with a high ratio between the metal halide and the ligands lose their ligands stepwise upon heating and transform into new compounds that usually show condensed metal–halide substructures (Näther *et al.*, 2001 ▸, 2002 ▸; Näther & Jess, 2004 ▸).

**Figure 1:**
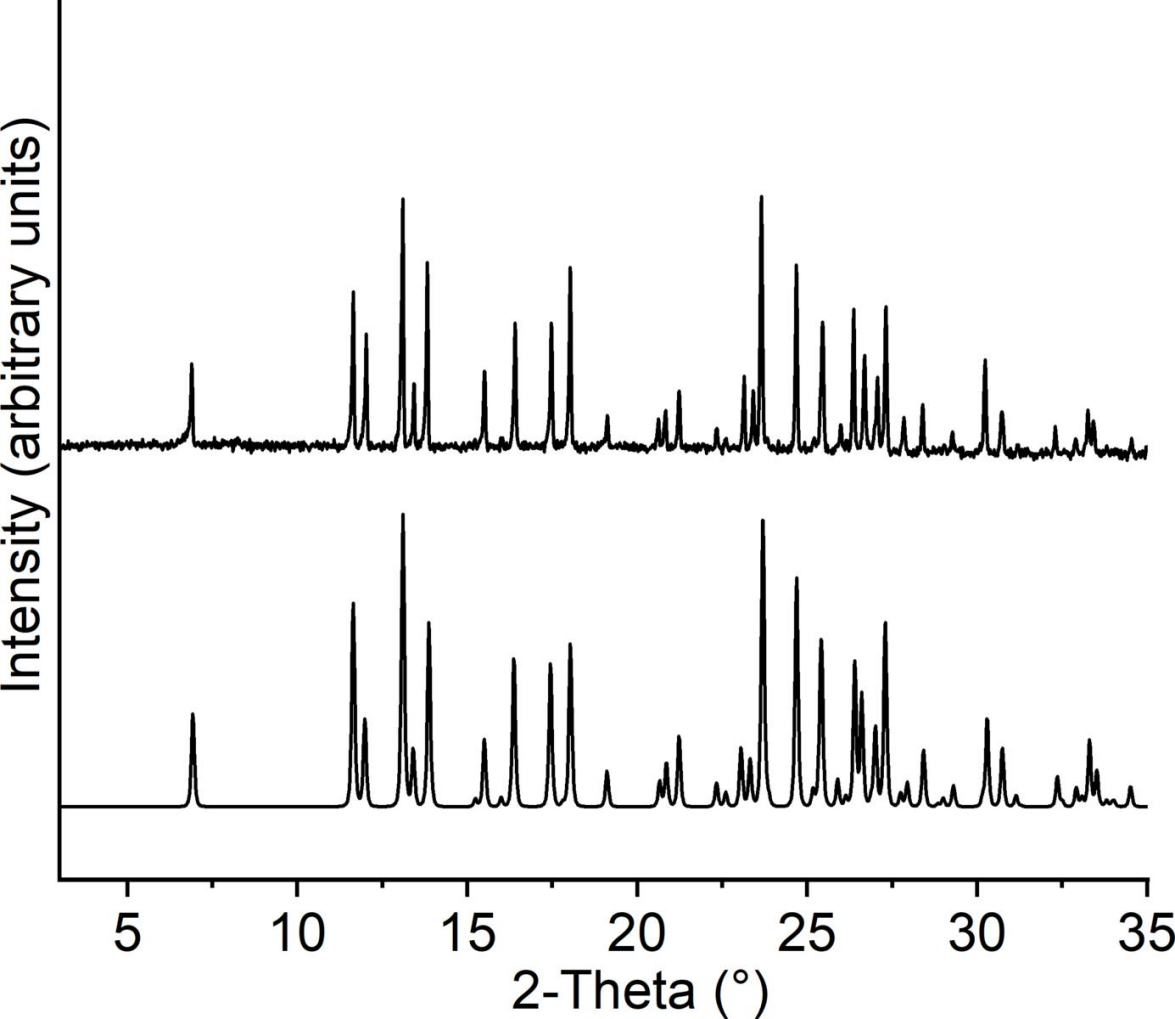
Experimental (top) and calculated (bottom) powder patterns for the title compound.

In this context, we have recently reported a new dinuclear complex with the composition [(CoBr_2_)_2_(2-methyl­pyridine *N*-oxide)_4_]·*n*-butanol in which the Co^II^ cations are fivefold coordinated by two bromide anions and one terminal as well as two bridging 2-methyl­pyridine *N*-oxide ligands and linked into dinuclear units by two symmetry-related μ-1,1(*O*,*O*) 2-methyl­pyridine *N*-oxide coligands (Näther & Jess, 2023 ▸). The *n*-butanol solvate mol­ecules can be removed by thermogravimetry, leading to the formation of a crystalline compound with the composition [CoBr_2_(2-methyl­pyridine *N*-oxide)_2_], for which the powder pattern is completely different from that of the pristine compound. We also found that the butanol mol­ecules have already been lost upon storage at room-temperature, leading to the same crystalline phase as that obtained by thermal ligand removal. Moreover, the new crystalline phase shows two endothermic events before decomposition, which points to an inter­esting thermal behavior. Unfortunately, we were not able to solve its structure from PXRD data. Therefore, in the present work we performed a large number of crystallization experiments. The crystals obtained were characterized by single-crystal X-ray diffraction. The analysis proves that a new compound with the composition [CoBr_2_(2-methyl­pyridine *N*-oxide)_2_] was obtained, consisting of discrete complexes for which the calculated powder pattern is identical to that of the phase obtained by butanol removal from the dinuclear complex mentioned above. Larger amounts of a crystalline powder are easily available and comparison of the experimental powder pattern with that calculated from single crystal data proves that the title compound was obtained as a pure phase (Fig. 1 ▸), which allowed a detailed investigation of the thermal properties of the title compound to be undertaken. 
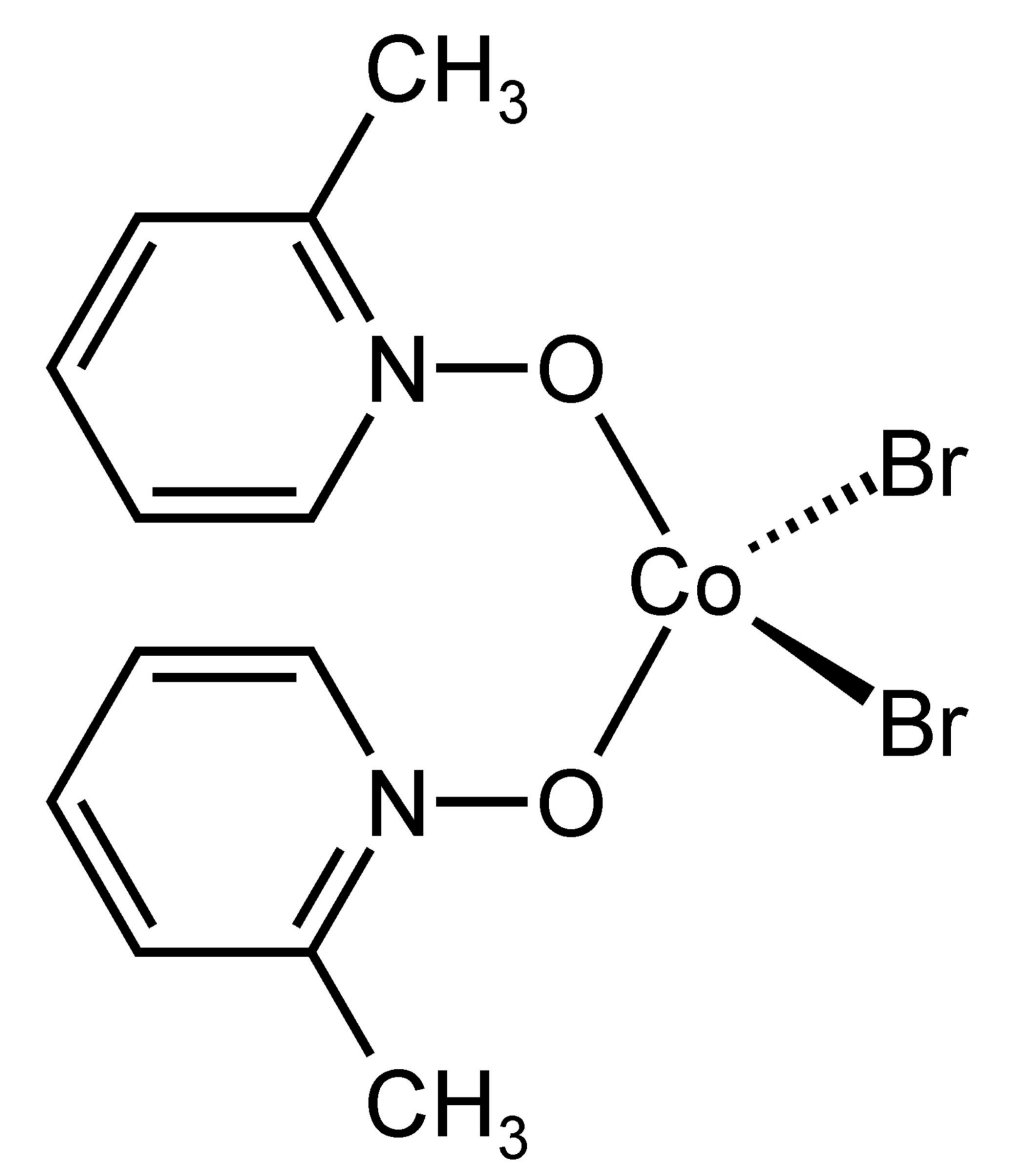


## 2. Structural commentary

**Figure 2:**
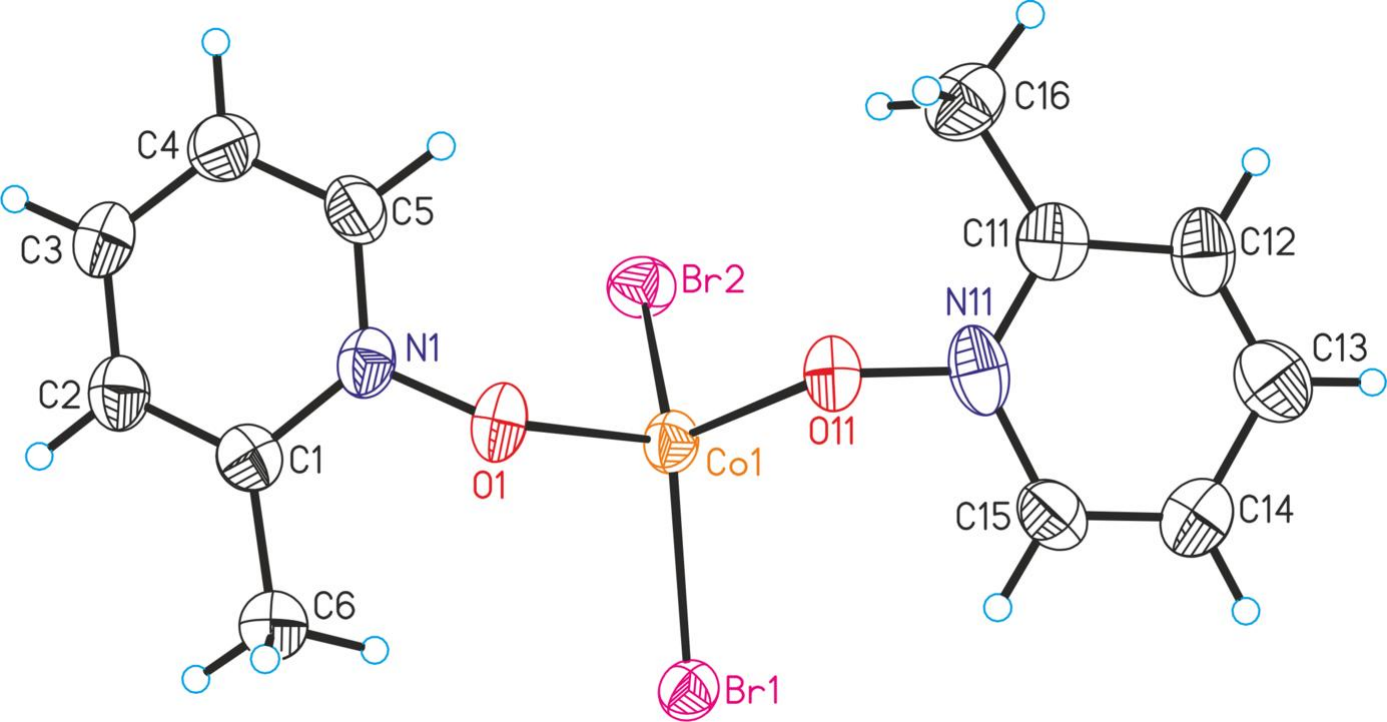
Crystal structure of the title compound with labeling and displacement ellipsoids drawn at the 50% probability level.

The asymmetric unit of the title compound, [CoBr_2_(2-methyl­pyridine *N*-oxide)_2_] (**1**), consists of one Co^II^ cation, two bromide anions and two 2-methyl­pyridine *N*-oxide coligands that are located in general positions (Fig. 2 ▸). Compound **1** forms discrete complexes in which the Co^II^ cations are fourfold coordinated by two bromide anions and two neutral 2-methyl­pyridine *N*-oxide coligands (Fig. 2 ▸). Bond lengths and angles correspond to literature values and show that the tetra­hedra are slightly distorted (Table 1 ▸). It is noted that the two known discrete tetra­hedral complexes [CuCl_2_(2-methyl­pyridine *N*-oxide)_2_] and [ZnCl_2_(2-methyl­pyridine *N*-oxide)_2_] (refcodes QQQBVY and QQQBXY; Kidd *et al.*, 1967 ▸) are not isotypic to the title compound. For the latter compound, this is surprising because there are many examples in the literature where tetra­hedral Co^II^ and Zn^II^ complexes are isotypic. On the other hand, there are very few examples reported in the literature where the thermodynamic relations between such complexes were fully investigated. It has been found, for example, that for two isotypic complexes the Co complex is thermodynamically stable at room temperature, whereas the isotypic Zn complex is metastable (Neumann *et al.*, 2018 ▸, 2019 ▸).

## 3. Supra­molecular features

**Figure 3:**
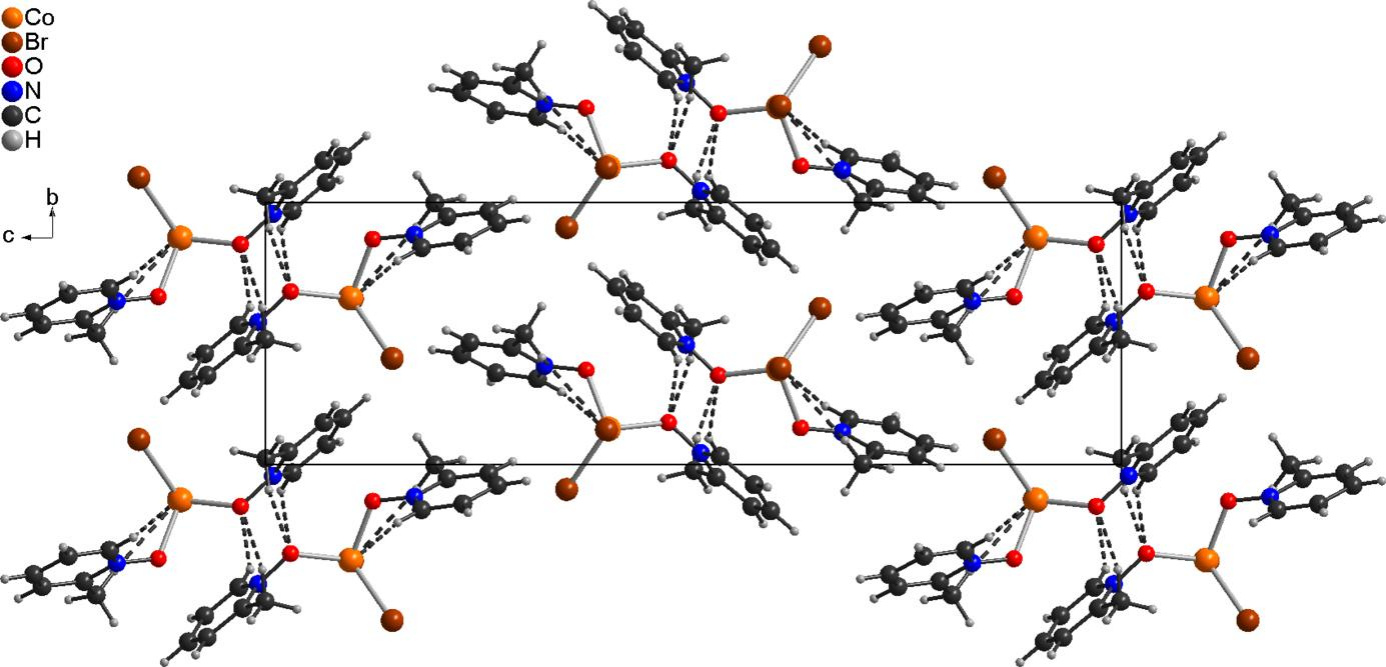
Crystal structure of the title compound viewed along the *a*-axis. Inter­molecular C—H⋯O and C—H⋯Br hydrogen bonds are shown as dashed lines.

In the crystal structure of compound **1**, a number of inter­molecular C—H⋯O and C—H⋯Br contacts are observed, but most of the contacts show angles far from linearity, indicating that these correspond to very weak inter­actions (Table 2 ▸). However, a few of them exhibit distances and angles that point to inter­molecular hydrogen bonding and, if they are considered as significant inter­actions, the discrete complexes are connected into chains propagating along the *a*-axis direction (Fig. 3 ▸ and Table 2 ▸).

## 4. Thermoanalytical investigations

As mentioned above, recent investigations of the dinuclear complex tetra­bromo-tetra­kis­(2-methyl­pyridine *N*-oxide)dicobalt(II) butanol solvate with thermogravimetry and differential thermoanalysis (TG-DTA) showed an endothermic signal after butanol removal where the sample mass did not change (Näther & Jess, 2023 ▸). Because it is the title complex that formed after solvent removal, its thermal properties were investigated in more detail using TG-DTA and DSC measurements (differential scanning calorimetry) as well as thermomicroscopy.

**Figure 4:**
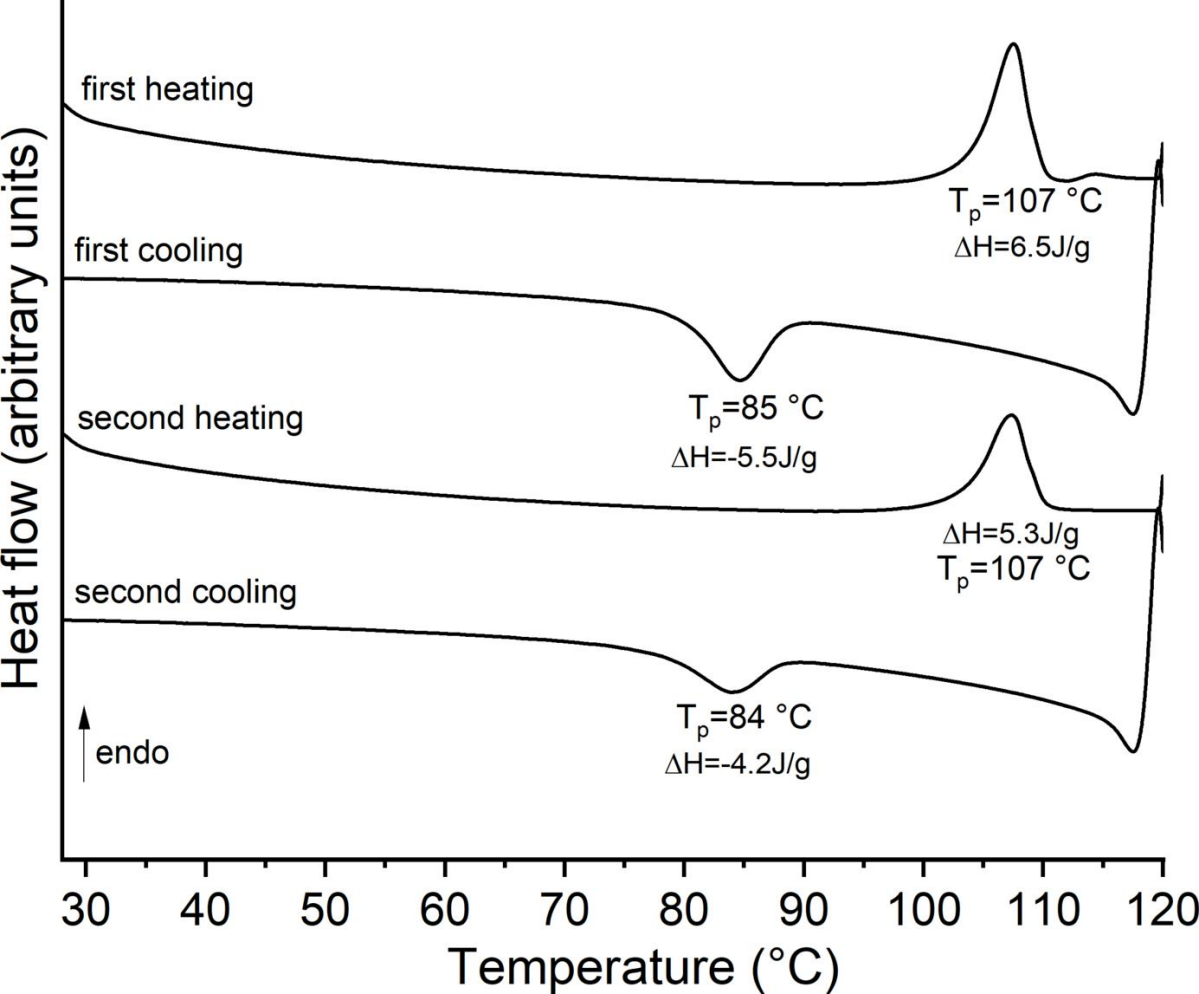
DSC heating and cooling runs for the title compound.

Upon heating, one poorly resolved mass loss is observed in the TG curve, which is accompanied by a strong exothermic event in the DTA curve at 278°C. The latter signal points to a decomposition of the 2-methyl­pyridine *N*-oxide ligand (Fig. S1). More importantly, before the first mass loss, two endothermic events at 109 and 155°C are observed in the DTA curve, which show that the overall thermal behavior is more complex. Therefore, DSC heating and cooling curves were measured, where two endothermic signals were observed upon heating (Fig. S2). Upon cooling, no exothermic signal was observed, which proves that the second endothermic event is irreversible. In contrast, if the title compound is measured up to 120°C and cooled down, an exothermic event is visible, which shows that this process is in principle reversible (Fig. 4 ▸). The same observations were made in the second heating and cooling run. However, the enthalpy of these events continuously decreases, which means that this event is not entirely reversible.

The residues obtained at 120 and 180°C in the DSC measurements were investigated by powder X-ray diffraction (PXRD), which showed that the residue formed after the first endothermic event corresponds to the title complex (Fig. S3). No PXRD pattern could be measured for the residue formed after the second endothermic event because it adhered to the bottom of the crucible, indicative of melting. To investigate this in more detail, thermomicroscopic measurements were performed, which show melting at about 164°C (Fig. S5). This is in agreement with other tetra­hedral Co but also Zn complexes, which melt upon heating (Neumann *et al.*, 2018 ▸, 2019 ▸).

Finally, to investigate the origin of the first reversible endothermic event at 109°C, single-crystal measurements were performed between 23 and 167°C. Surprisingly, there are no structural changes and all data sets could be refined perfectly in space group *P*2_1_2_1_2_1_. The crystal decomposes upon further heating. The reason for this thermal event is therefore still unknown.

## 5. Database survey

A search of the CSD (version 5.43, last update March 2023; Groom *et al.*, 2016 ▸) using CONQUEST (Bruno *et al.*, 2002 ▸) reveals that no crystal structures of cobalt halide compounds with 2-methyl­pyridine *N*-oxide have been reported. As mentioned above, one compound with the composition [(CoBr_2_)_2_(2-methyl­pyridine *N*-oxide)_4_]·*n*-butanol was published recently (Näther & Jess, 2023 ▸) but does not yet appear as a hit.

For CuCl_2_ and ZnCl_2_, two discrete tetra­hedral complexes with the composition [CuCl_2_(2-methyl­pyridine *N*-oxide)_2_] and [ZnCl_2_(2-methyl­pyridine *N*-oxide)_2_] have been reported, but neither of them is isotypic to the title compound (refcodes QQQBVY and QQQBXY; Kidd, *et al.*, 1967 ▸). Similar complexes with a tetra­hedral coordination are also reported with CuCl_2_ and ZnCl_2_ and 3-methyl­pyridine *N*-oxide and 4-methyl­pyridine, respectively, as ligands [QQQBWA, QQQBWA01, QQQBXM (Kidd *et al.*, 1967 ▸), CMPOCU (Watson & Johnson, 1971 ▸), and CMPOCU01, QQQBXG (Kidd *et al.*, 1967 ▸)]. Finally, [ZnI_2_(4-methyl­pyridine *N*-oxide)_2_] also forms a tetra­hedral complex (SANRUV; Shi *et al.*, 2005 ▸).

There are additional compounds with different structures and 2-methyl­pyridine *N*-oxide as ligand, including [(CuCl_2_)_3_(2-methyl­pyridine *N*-oxide)_2_(H_2_O)_2_] (PIOCUA; Sager & Watson, 1968 ▸), [MnCl_2_(2-methyl­pyridine *N*-oxide)(H_2_O)] (VEJMAB; Kang *et al.*, 2017 ▸), and [(MnBr_2_)_2_(2-methyl­pyridine *N*-oxide)_2_(H_2_O)_4_] bis­(2-methyl­pyridine *N*-oxide) solvate (VONHEO; Lynch *et al.*, 2019 ▸).

Lastly, 2-methyl­pyridine *N*-oxide in its protonated cationic form together with a tetra­chloro aurate(III) anion and a neutral 2-methyl­pyridine *N*-oxide (CICBIZ; Hussain & Aziz Al-Hamoud, 1984 ▸) and Co(2-methyl­pyridine *N*-oxide)_5_ with two ClO_4_^−^ counter-ions [PICOCO (Coyle & Ibers, 1970 ▸) and PICOCO01 (Bertini *et al.*, 1975 ▸)] have been reported.

## 6. Synthesis and crystallization

CoBr_2_ (97%) was purchased from Alfa Aesar and 2-methyl­pyridine *N*-oxide (98%) was obtained from Thermo Scientific.

**Synthesis:**

109 mg CoBr_2_ (0.5mmol) and 218 mg 2-picoline *N*-oxide (2 mmol) were stirred for 1 d in *n*-butanol at room temperature. The precipitate was filtered off and dried in air. Single crystals were obtained using the same conditions but without stirring. N.B. When stoichiometric amounts were used, in some batches a very small amount of the known compound [CoBr_2_]_2_(2-methyl­pyridine *N*-oxide)_4_·*n*-butanol was found (Näther & Jess, 2023 ▸). The IR spectrum of the title compound is shown in Fig. S5.

**Experimental details:**

The PXRD measurements were performed with a Stoe Transmission Powder Diffraction System (STADI P) equipped with a MYTHEN 1K detector and a Johansson-type Ge(111) monochromator using Cu *K*α_1_ radiation (λ = 1.540598 Å). Thermogravimetry and differential thermoanalysis (TG-DTA) measurements were performed in a dynamic nitro­gen atmosphere in Al_2_O_3_ crucibles using a STA-PT 1000 thermobalance from Linseis. The instrument was calibrated using standard reference materials. Differential scanning calorimetry measurements were performed with a DSC from Mettler Toledo in Al pans under nitro­gen atmosphere with 10°C min^−1^. The IR spectra were measured using an ATI Mattson Genesis Series FTIR Spectrometer, control software: *WINFIRST*, from ATI Mattson.

## 7. Refinement

Crystal data, data collection and structure refinement details are summarized in Table 3 ▸. C-bound hydrogen atoms were positioned with idealized geometry (methyl H atoms allowed to rotate but not to tip) and were refined isotropically with *U*_ĩso_(H) = 1.2 *U*_eq_(C) (1.5 for methyl hydrogen atoms) using a riding model. One reflection (outlier) was removed using the OMIT command.

## Supplementary Material

Crystal structure: contains datablock(s) I. DOI: 10.1107/S2056989024000252/yz2048sup1.cif

Structure factors: contains datablock(s) I. DOI: 10.1107/S2056989024000252/yz2048Isup2.hkl

DTG, TG and DTA curve for the title compound. DOI: 10.1107/S2056989024000252/yz2048sup3.png

DSC heating and cooling curve of the title compound measured to 180C with 10C/min. DOI: 10.1107/S2056989024000252/yz2048sup4.png

Experimental powder pattern of the residue obtained at 120C in a DSC measurement of the title compound. DOI: 10.1107/S2056989024000252/yz2048sup5.png

Microscopic images of the title compound at different temperatures. DOI: 10.1107/S2056989024000252/yz2048sup6.jpg

IR spectrum of the title compound. The values of the most prominent vibrations are given. DOI: 10.1107/S2056989024000252/yz2048sup7.png

CCDC reference: 2324135

Additional supporting information:  crystallographic information; 3D view; checkCIF report

## Figures and Tables

**Table 1:** Selected geometric parameters (Å, °)

Co1—Br1	2.3874 (11)	Co1—O1	1.973 (5)
Co1—Br2	2.3951 (11)	Co1—O11	1.954 (4)
			
Br1—Co1—Br2	112.83 (4)	O11—Co1—Br1	114.62 (14)
O1—Co1—Br1	108.39 (15)	O11—Co1—Br2	112.76 (15)
O1—Co1—Br2	111.40 (14)	O11—Co1—O1	95.48 (18)

**Table 2:** Hydrogen-bond geometry (Å, °)

*D*—H⋯*A*	*D*—H	H⋯*A*	*D*⋯*A*	*D*—H⋯*A*
C2—H2⋯Br1^i^	0.95	3.06	3.744 (7)	131
C2—H2⋯Br2^i^	0.95	3.13	3.884 (7)	137
C5—H5⋯Br1^ii^	0.95	2.91	3.806 (7)	158
C6—H6*A*⋯Br1	0.98	3.03	3.999 (7)	172
C14—H14⋯Br2^iii^	0.95	3.12	3.754 (8)	126
C14—H14⋯O1^iv^	0.95	2.48	3.160 (9)	129
C15—H15⋯O11^iv^	0.95	2.50	3.358 (8)	150
C16—H16*C*⋯O11^v^	0.98	2.45	3.368 (9)	157

Symmetry codes: (i) 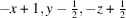 ; (ii) 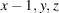 ; (iii) 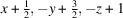 ; (iv) 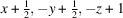 ; (v) 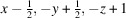 .

**Table 3:** Experimental details

Crystal data	
Chemical formula	[CoBr_2_(C_6_H_7_NO)_2_]
*M*_r_	437.00
Crystal system, space group	Orthorhombic, *P*2_1_2_1_2_1_
Temperature (K)	100
*a*, *b*, *c* (Å)	7.6106 (2), 7.8024 (2), 25.4699 (5)
*V* (Å^3^)	1512.43 (6)
*Z*	4
Radiation type	Cu *K*α
μ (mm^−1^)	15.09
Crystal size (mm)	0.18 × 0.04 × 0.03

Data collection	
Diffractometer	XtaLAB Synergy, Dualflex, HyPix
Absorption correction	Multi-scan (*CrysAlis PRO*; Rigaku OD, 2022 ▸)
*T*_min_, *T*_max_	0.448, 1.000
No. of measured, independent and observed [*I* > 2σ(*I*)] reflections	9313, 3235, 3183
*R*_int_	0.030
(sin θ/λ)_max_ (Å^−1^)	0.640

Refinement	
*R*[*F* ^2^ > 2σ(*F* ^2^)], *wR*(*F* ^2^), *S*	0.036, 0.093, 1.06
No. of reflections	3235
No. of parameters	174
H-atom treatment	H-atom parameters constrained
Δρ_max_, Δρ_min_ (e Å^−3^)	0.65, −0.47
Absolute structure	Flack *x* determined using 1248 quotients [(*I* ^+^)−(*I* ^−^)]/[(*I* ^+^)+(*I* ^−^)] (Parsons *et al.*, 2013 ▸)
Absolute structure parameter	−0.026 (5)

Computer programs: *CrysAlis PRO* (Rigaku OD, 2022 ▸), *SHELXT2014/4* (Sheldrick, 2015*a* ▸), *SHELXL2016/6* (Sheldrick, 2015*b* ▸), *DIAMOND* (Brandenburg & Putz, 1999 ▸) and *XP* in *SHELXTL-PC* (Sheldrick, 2008 ▸) and *publCIF* (Westrip, 2010 ▸).

## supplementary crystallographic information

### Crystal data

**Table:**

[CoBr_2_(C_6_H_7_NO)_2_]	*D*_x_ = 1.919 Mg m^−^^3^
*M**_r_* = 437.00	Cu *K*α radiation, λ = 1.54184 Å
Orthorhombic, *P*2_1_2_1_2_1_	Cell parameters from 7293 reflections
*a* = 7.6106 (2) Å	θ = 3.5–78.6°
*b* = 7.8024 (2) Å	µ = 15.09 mm^−^^1^
*c* = 25.4699 (5) Å	*T* = 100 K
*V* = 1512.43 (6) Å^3^	Needle, blue
*Z* = 4	0.18 × 0.04 × 0.03 mm
*F*(000) = 852	

### Data collection

**Table:**

XtaLAB Synergy, Dualflex, HyPix diffractometer	3235 independent reflections
Radiation source: micro-focus sealed X-ray tube, PhotonJet (Cu) X-ray Source	3183 reflections with *I* > 2σ(*I*)
Mirror monochromator	*R*_int_ = 0.030
Detector resolution: 10.0000 pixels mm^-1^	θ_max_ = 80.5°, θ_min_ = 3.5°
ω scans	*h* = −9→9
Absorption correction: multi-scan (CrysAlisPro; Rigaku OD, 2022)	*k* = −9→7
*T*_min_ = 0.448, *T*_max_ = 1.000	*l* = −32→31
9313 measured reflections	

### Refinement

**Table:**

Refinement on *F*^2^	Hydrogen site location: inferred from neighbouring sites
Least-squares matrix: full	H-atom parameters constrained
*R*[*F*^2^ > 2σ(*F*^2^)] = 0.036	*w* = 1/[σ^2^(*F*_o_^2^) + (0.0482*P*)^2^ + 3.0792*P*] where *P* = (*F*_o_^2^ + 2*F*_c_^2^)/3
*wR*(*F*^2^) = 0.093	(Δ/σ)_max_ = 0.001
*S* = 1.06	Δρ_max_ = 0.65 e Å^−^^3^
3235 reflections	Δρ_min_ = −0.47 e Å^−^^3^
174 parameters	Absolute structure: Flack *x* determined using 1248 quotients [(*I*^+^)-(*I*^-^)]/[(*I*^+^)+(*I*^-^)] (Parsons *et al.*, 2013)
0 restraints	Absolute structure parameter: −0.026 (5)
Primary atom site location: dual	

### Special details

**Table:**

Geometry. All esds (except the esd in the dihedral angle between two l.s. planes) are estimated using the full covariance matrix. The cell esds are taken into account individually in the estimation of esds in distances, angles and torsion angles; correlations between esds in cell parameters are only used when they are defined by crystal symmetry. An approximate (isotropic) treatment of cell esds is used for estimating esds involving l.s. planes.

### Fractional atomic coordinates and isotropic or equivalent isotropic displacement parameters (Å^2^)

**Table:**

	*x*	*y*	*z*	*U*_iso_*/*U*_eq_	
Co1	0.42209 (13)	0.36450 (12)	0.40064 (4)	0.0302 (2)	
Br1	0.73569 (8)	0.36730 (8)	0.39846 (2)	0.03254 (16)	
Br2	0.29398 (9)	0.59533 (9)	0.35188 (3)	0.04006 (18)	
O1	0.3404 (6)	0.1401 (6)	0.37462 (16)	0.0350 (9)	
N1	0.2739 (7)	0.1236 (7)	0.32603 (18)	0.0320 (10)	
C1	0.3749 (9)	0.0537 (8)	0.2878 (3)	0.0337 (13)	
C2	0.3008 (10)	0.0343 (9)	0.2389 (3)	0.0380 (14)	
H2	0.369838	−0.011608	0.211185	0.046*	
C3	0.1283 (10)	0.0798 (10)	0.2291 (3)	0.0401 (15)	
H3	0.078032	0.062728	0.195331	0.048*	
C4	0.0306 (10)	0.1505 (10)	0.2693 (3)	0.0410 (15)	
H4	−0.087882	0.183703	0.263439	0.049*	
C5	0.1060 (9)	0.1723 (9)	0.3177 (3)	0.0375 (14)	
H5	0.039908	0.221735	0.345488	0.045*	
C6	0.5573 (10)	0.0018 (9)	0.3022 (3)	0.0405 (15)	
H6A	0.612177	0.092720	0.323040	0.061*	
H6B	0.625929	−0.017063	0.270125	0.061*	
H6C	0.553525	−0.104245	0.322723	0.061*	
O11	0.3208 (6)	0.3399 (6)	0.47074 (16)	0.0345 (9)	
N11	0.3415 (9)	0.4564 (7)	0.5087 (2)	0.0406 (14)	
C11	0.2092 (11)	0.5484 (9)	0.5266 (3)	0.0414 (15)	
C12	0.2428 (12)	0.6598 (8)	0.5683 (3)	0.0441 (17)	
H12	0.148608	0.726718	0.581688	0.053*	
C13	0.3997 (12)	0.6768 (10)	0.5901 (3)	0.0497 (18)	
H13	0.417117	0.755090	0.618185	0.060*	
C14	0.5407 (11)	0.5771 (10)	0.5711 (3)	0.0460 (17)	
H14	0.653938	0.586838	0.586508	0.055*	
C15	0.5133 (10)	0.4686 (10)	0.5312 (3)	0.0406 (15)	
H15	0.606829	0.400540	0.518027	0.049*	
C16	0.0416 (10)	0.5222 (10)	0.5006 (3)	0.0462 (17)	
H16A	0.052604	0.552188	0.463370	0.069*	
H16B	−0.047766	0.595112	0.516994	0.069*	
H16C	0.006869	0.401760	0.503838	0.069*	

### Atomic displacement parameters (Å^2^)

**Table:**

	*U*^11^	*U*^22^	*U*^33^	*U*^12^	*U*^13^	*U*^23^
Co1	0.0325 (5)	0.0338 (5)	0.0241 (4)	−0.0023 (4)	0.0002 (4)	−0.0012 (4)
Br1	0.0320 (3)	0.0355 (3)	0.0301 (3)	−0.0009 (2)	−0.0015 (2)	0.0013 (2)
Br2	0.0382 (3)	0.0437 (4)	0.0383 (3)	0.0048 (3)	−0.0011 (3)	0.0066 (3)
O1	0.046 (2)	0.035 (2)	0.0242 (19)	−0.007 (2)	−0.0032 (17)	−0.0020 (17)
N1	0.036 (2)	0.034 (2)	0.026 (2)	−0.008 (2)	0.0017 (19)	−0.0017 (19)
C1	0.035 (3)	0.033 (3)	0.033 (3)	−0.002 (2)	0.004 (2)	0.002 (2)
C2	0.045 (4)	0.039 (3)	0.030 (3)	−0.004 (3)	0.002 (3)	−0.002 (2)
C3	0.046 (4)	0.042 (4)	0.033 (3)	−0.006 (3)	−0.005 (3)	−0.003 (3)
C4	0.037 (3)	0.042 (4)	0.044 (4)	0.000 (3)	−0.003 (3)	−0.001 (3)
C5	0.037 (3)	0.036 (3)	0.039 (3)	0.000 (3)	0.007 (3)	−0.010 (3)
C6	0.040 (4)	0.042 (4)	0.039 (4)	0.002 (3)	−0.002 (3)	−0.003 (3)
O11	0.043 (2)	0.033 (2)	0.0273 (19)	−0.0040 (19)	0.0015 (18)	−0.0040 (17)
N11	0.064 (4)	0.030 (3)	0.028 (2)	−0.004 (3)	0.010 (3)	0.000 (2)
C11	0.048 (4)	0.037 (3)	0.039 (3)	−0.002 (3)	0.001 (3)	0.005 (3)
C12	0.065 (5)	0.032 (3)	0.035 (3)	−0.002 (3)	0.001 (3)	−0.002 (2)
C13	0.056 (5)	0.040 (4)	0.053 (4)	0.000 (3)	0.008 (4)	−0.003 (3)
C14	0.049 (4)	0.047 (4)	0.042 (4)	−0.004 (3)	−0.006 (3)	0.003 (3)
C15	0.045 (4)	0.040 (3)	0.037 (3)	0.010 (3)	0.007 (3)	0.002 (3)
C16	0.044 (4)	0.050 (4)	0.045 (4)	−0.012 (3)	−0.004 (3)	0.007 (3)

### Geometric parameters (Å, º)

**Table:**

Co1—Br1	2.3874 (11)	C6—H6B	0.9800
Co1—Br2	2.3951 (11)	C6—H6C	0.9800
Co1—O1	1.973 (5)	O11—N11	1.336 (7)
Co1—O11	1.954 (4)	N11—C11	1.318 (10)
O1—N1	1.343 (6)	N11—C15	1.431 (10)
N1—C1	1.355 (8)	C11—C12	1.396 (10)
N1—C5	1.350 (9)	C11—C16	1.452 (11)
C1—C2	1.376 (10)	C12—H12	0.9500
C1—C6	1.491 (10)	C12—C13	1.323 (12)
C2—H2	0.9500	C13—H13	0.9500
C2—C3	1.383 (10)	C13—C14	1.411 (12)
C3—H3	0.9500	C14—H14	0.9500
C3—C4	1.380 (10)	C14—C15	1.339 (11)
C4—H4	0.9500	C15—H15	0.9500
C4—C5	1.371 (10)	C16—H16A	0.9800
C5—H5	0.9500	C16—H16B	0.9800
C6—H6A	0.9800	C16—H16C	0.9800
			
Br1—Co1—Br2	112.83 (4)	H6A—C6—H6B	109.5
O1—Co1—Br1	108.39 (15)	H6A—C6—H6C	109.5
O1—Co1—Br2	111.40 (14)	H6B—C6—H6C	109.5
O11—Co1—Br1	114.62 (14)	N11—O11—Co1	123.2 (4)
O11—Co1—Br2	112.76 (15)	O11—N11—C15	116.3 (6)
O11—Co1—O1	95.48 (18)	C11—N11—O11	122.1 (7)
N1—O1—Co1	120.9 (4)	C11—N11—C15	121.5 (6)
O1—N1—C1	119.1 (5)	N11—C11—C12	117.6 (7)
O1—N1—C5	118.3 (5)	N11—C11—C16	115.8 (7)
C5—N1—C1	122.5 (5)	C12—C11—C16	126.6 (8)
N1—C1—C2	117.5 (6)	C11—C12—H12	118.4
N1—C1—C6	117.5 (6)	C13—C12—C11	123.1 (8)
C2—C1—C6	125.0 (6)	C13—C12—H12	118.4
C1—C2—H2	119.2	C12—C13—H13	120.4
C1—C2—C3	121.7 (7)	C12—C13—C14	119.2 (7)
C3—C2—H2	119.2	C14—C13—H13	120.4
C2—C3—H3	120.7	C13—C14—H14	120.3
C4—C3—C2	118.7 (6)	C15—C14—C13	119.3 (8)
C4—C3—H3	120.7	C15—C14—H14	120.3
C3—C4—H4	120.3	N11—C15—H15	120.4
C5—C4—C3	119.4 (7)	C14—C15—N11	119.3 (7)
C5—C4—H4	120.3	C14—C15—H15	120.4
N1—C5—C4	120.2 (6)	C11—C16—H16A	109.5
N1—C5—H5	119.9	C11—C16—H16B	109.5
C4—C5—H5	119.9	C11—C16—H16C	109.5
C1—C6—H6A	109.5	H16A—C16—H16B	109.5
C1—C6—H6B	109.5	H16A—C16—H16C	109.5
C1—C6—H6C	109.5	H16B—C16—H16C	109.5

### Hydrogen-bond geometry (Å, º)

**Table:**

*D*—H···*A*	*D*—H	H···*A*	*D*···*A*	*D*—H···*A*
C2—H2···Br1^i^	0.95	3.06	3.744 (7)	131
C2—H2···Br2^i^	0.95	3.13	3.884 (7)	137
C5—H5···Br1^ii^	0.95	2.91	3.806 (7)	158
C6—H6*A*···Br1	0.98	3.03	3.999 (7)	172
C14—H14···Br2^iii^	0.95	3.12	3.754 (8)	126
C14—H14···O1^iv^	0.95	2.48	3.160 (9)	129
C15—H15···O11^iv^	0.95	2.50	3.358 (8)	150
C16—H16*C*···O11^v^	0.98	2.45	3.368 (9)	157

Symmetry codes: (i) −*x*+1, *y*−1/2, −*z*+1/2; (ii) *x*−1, *y*, *z*; (iii) *x*+1/2, −*y*+3/2, −*z*+1; (iv) *x*+1/2, −*y*+1/2, −*z*+1; (v) *x*−1/2, −*y*+1/2, −*z*+1.
