# Supplementary figures and images for: Synthesis, crystal structure and thermal properties of di­bromido­bis­(2-methyl­pyridine N-oxide-κO)cobalt(II)
Source: Acta Crystallogr E Crystallogr Commun. 2024 Jan 12;80(Pt 2):152–6. doi: 10.1107/S2056989024000252 (PMC10848989; doi:10.1107/S2056989024000252)

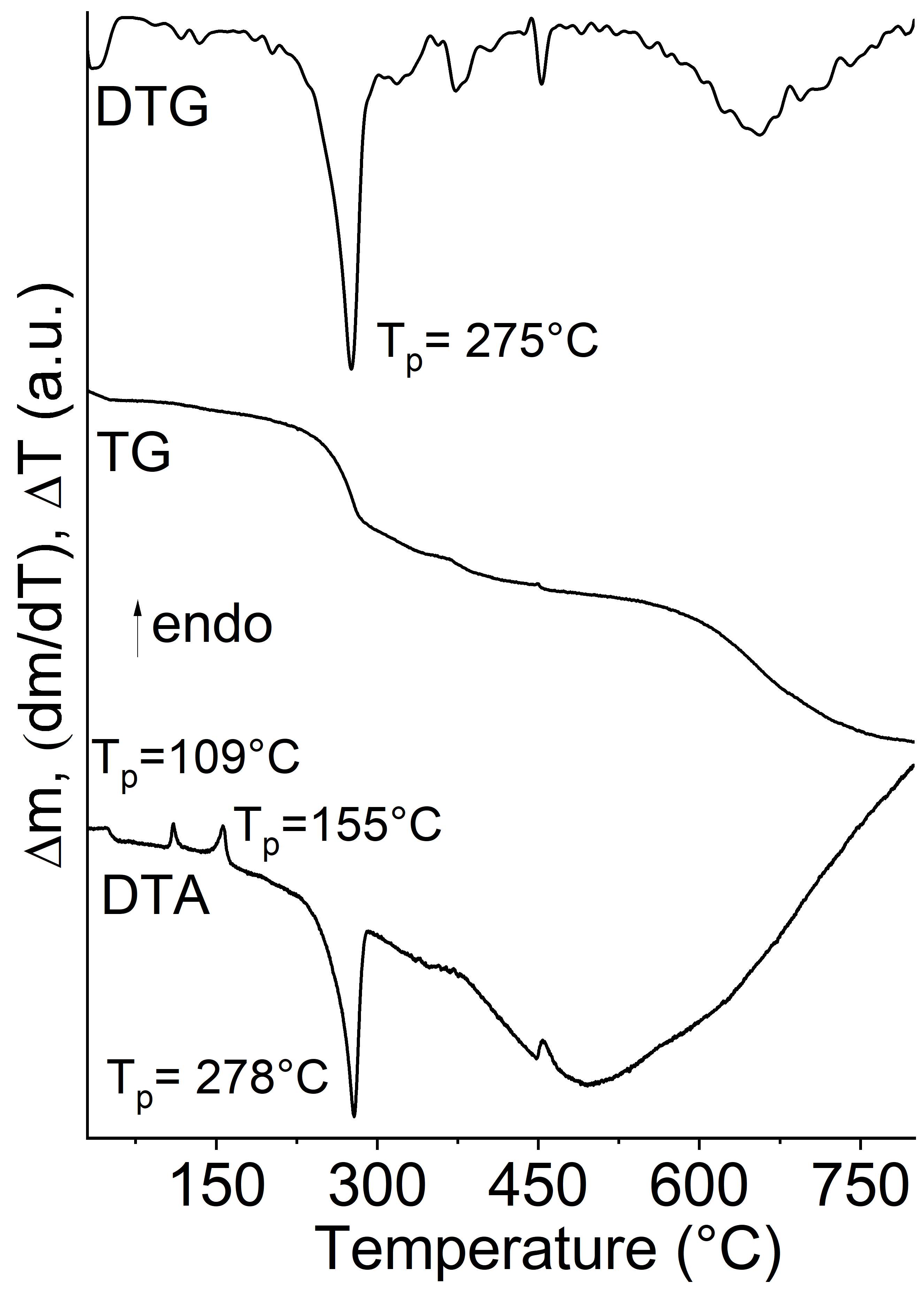

Supplement: Supplementary file 3 [file e-80-00152-sup3.png]

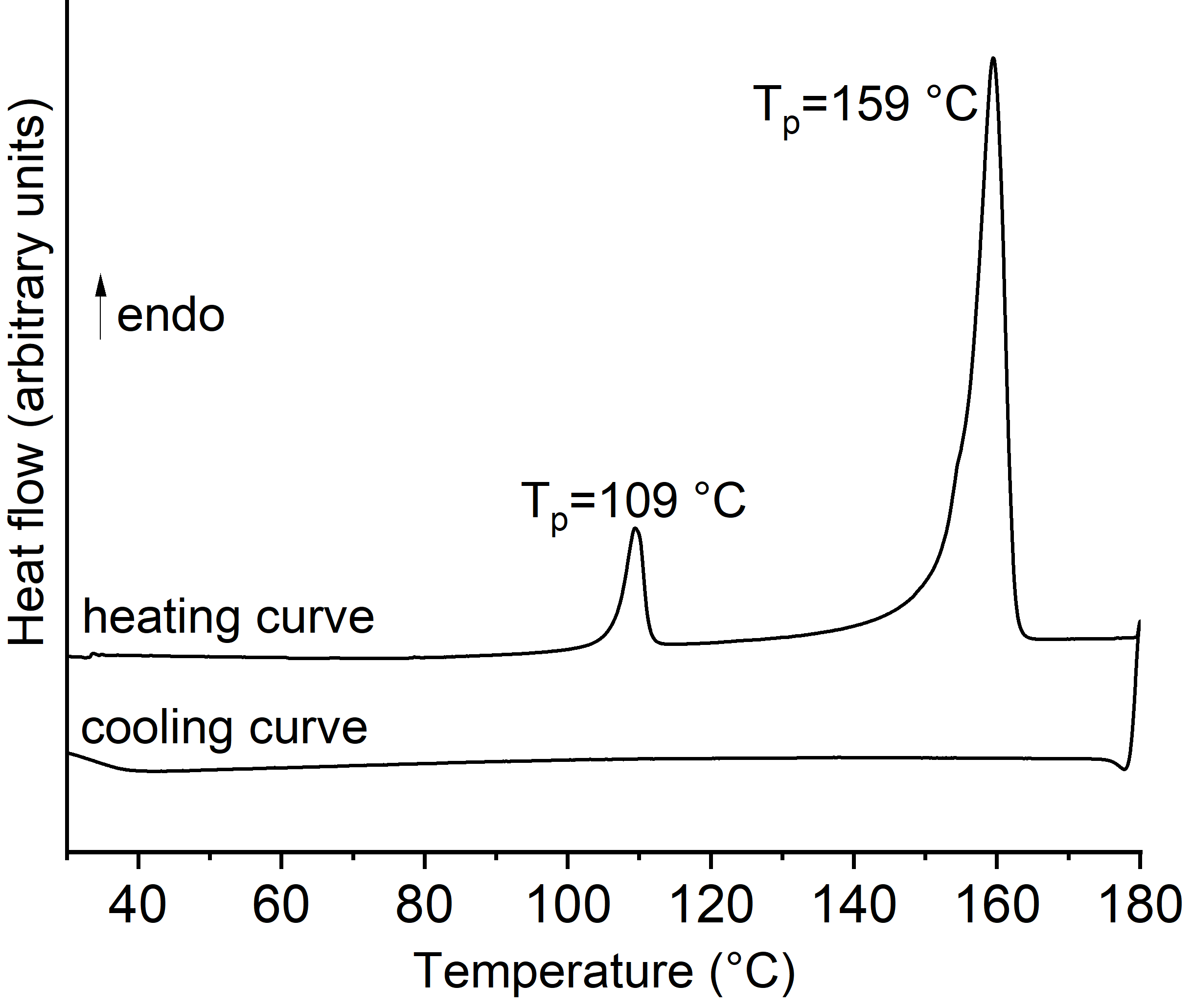

Supplement: Supplementary file 4 [file e-80-00152-sup4.png]

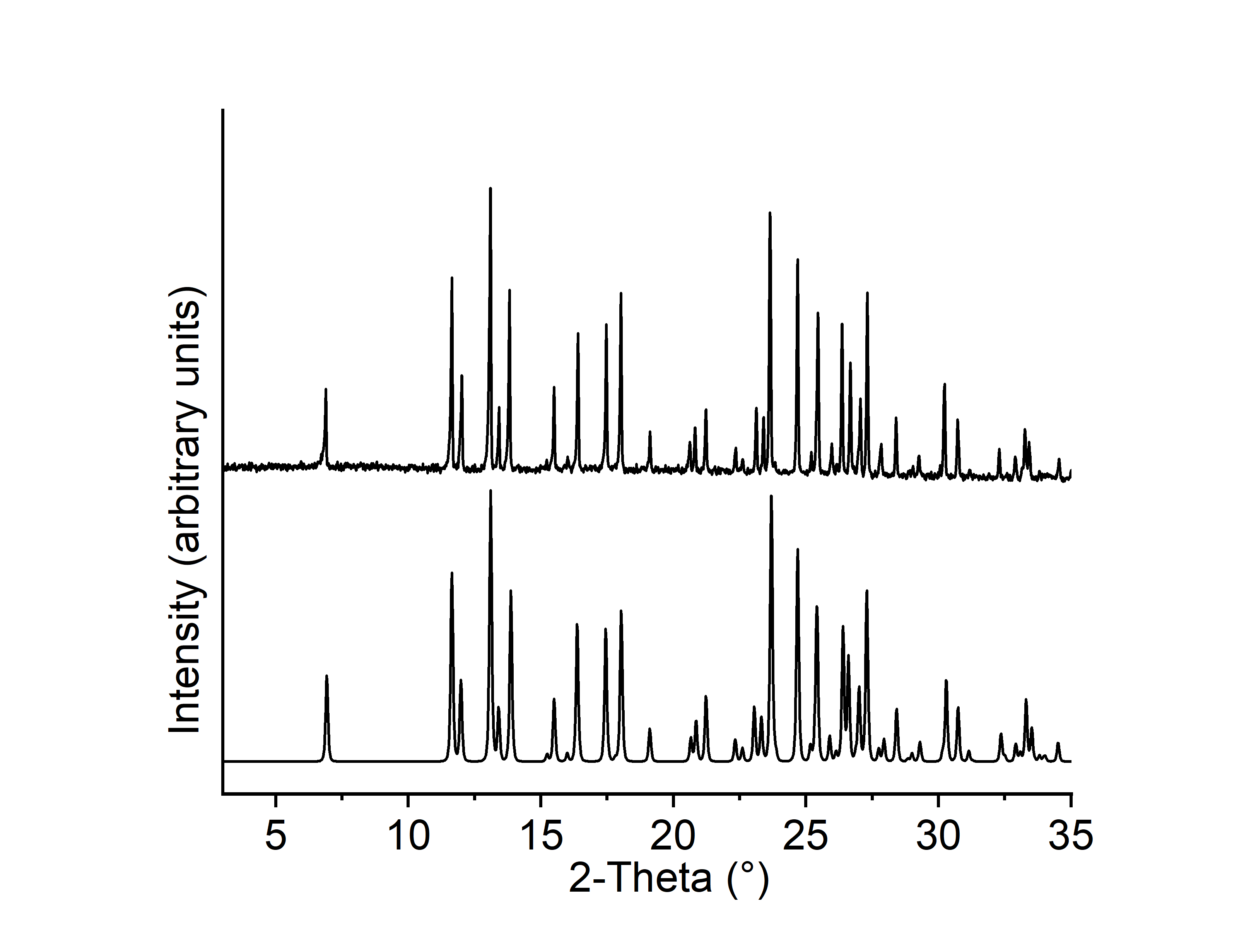

Supplement: Supplementary file 5 [file e-80-00152-sup5.png]

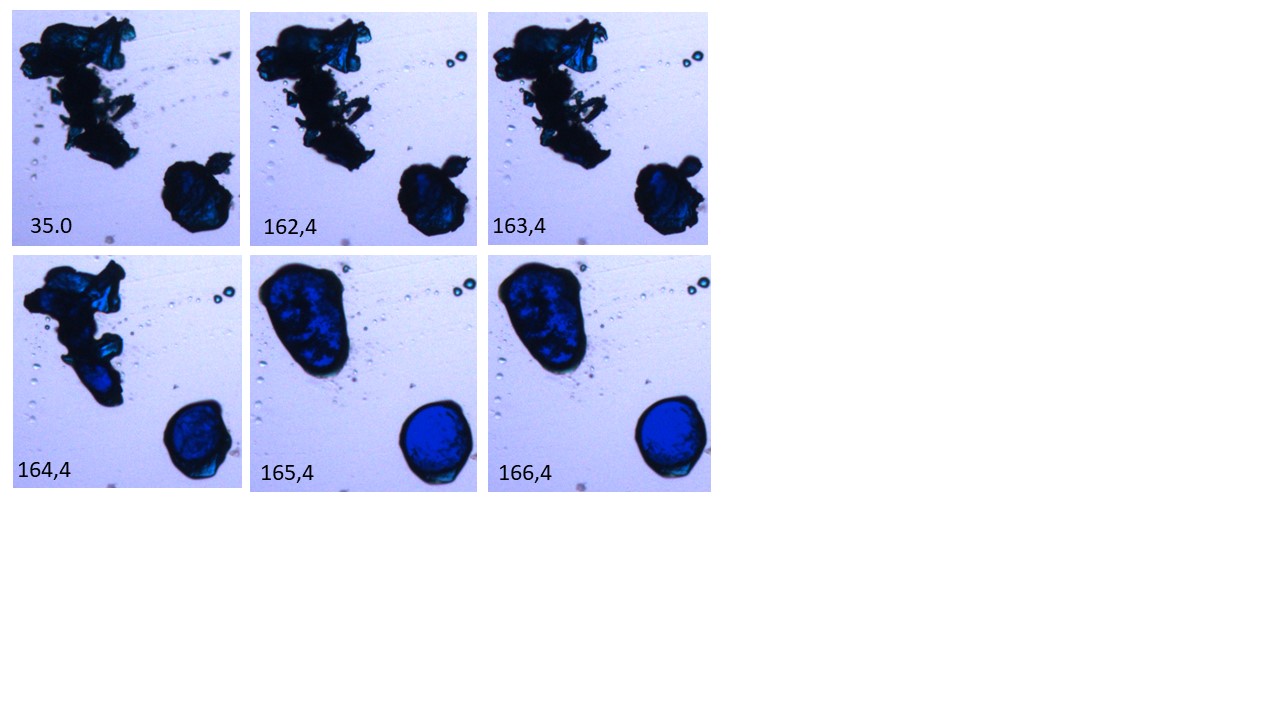

Supplement: Supplementary file 6 [file e-80-00152-sup6.jpg]

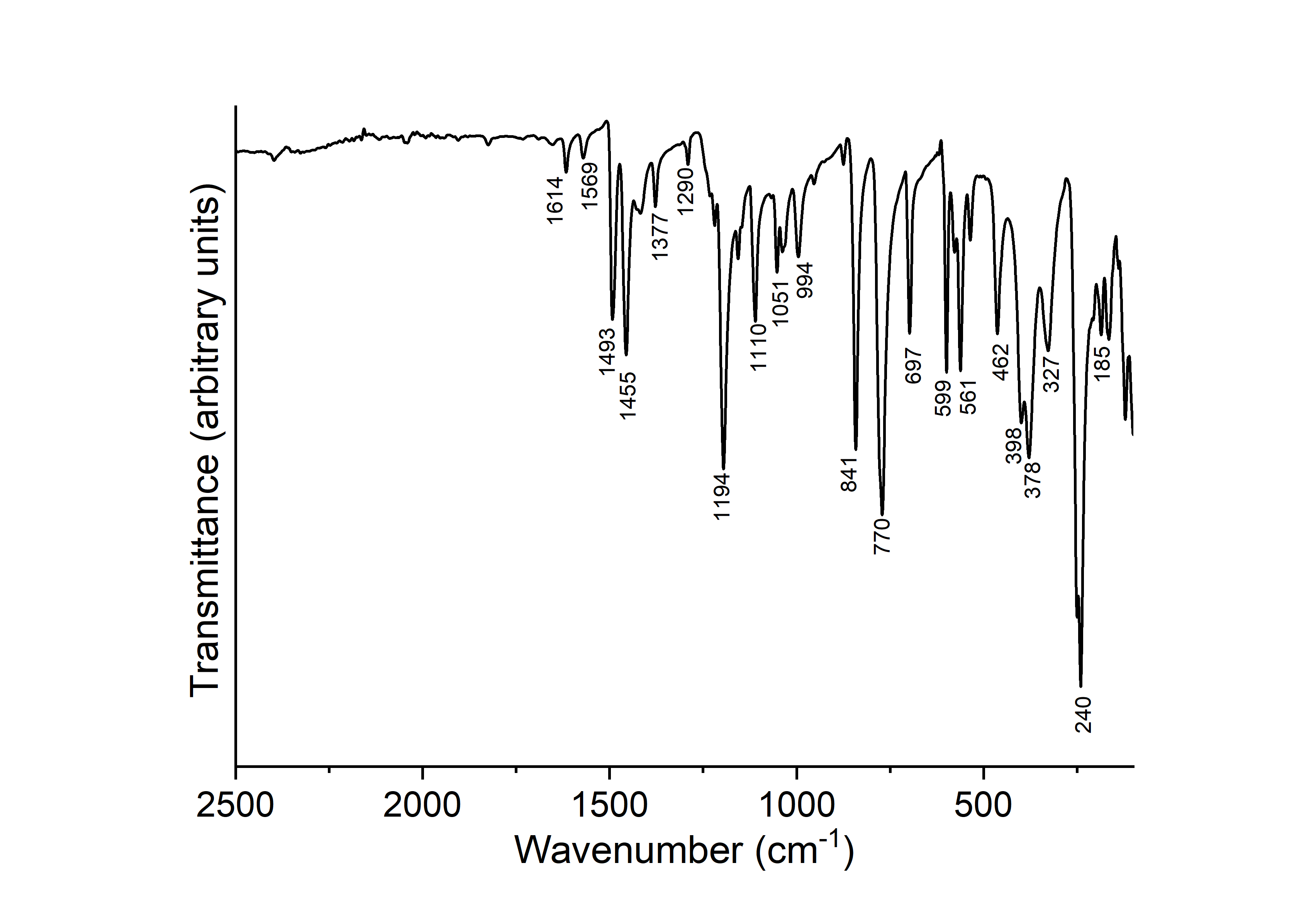

Supplement: Supplementary file 7 [file e-80-00152-sup7.png]
